# Delayed Turnover of Unphosphorylated Ssk1 during Carbon Stress Activates the Yeast Hog1 Map Kinase Pathway

**DOI:** 10.1371/journal.pone.0137199

**Published:** 2015-09-04

**Authors:** Milene Carmes Vallejo, Peter Mayinger

**Affiliations:** Division of Nephrology & Hypertension, Oregon Health & Science University, Portland, Oregon, 97239, United States of America; NIEHS/NIH, UNITED STATES

## Abstract

In *Saccharomyces cerevisiae*, the Hog1 mitogen-activated protein kinase (MAPK) pathway coordinates the adaptation to osmotic stress and was recently reported to respond to acute changes in glucose levels. Similarly as in osmotic stress, glucose starvation leads to a transient accumulation of Hog1 in the nucleus. However, the kinetics and the mechanism of Hog1 activation are different for these stress conditions. During osmotic shock the activation of Hog1 can be transduced by either the Sho1 or the Sln1/Ypd1/Ssk1 branch. During glucose starvation the phosphorylation of Hog1 is slower and is completely dependent on Ssk1, but independent of Sho1. To characterize the mechanism of activation of Hog1 during carbon stress, we examined the turnover of Ssk1 protein levels upon glucose starvation in the presence of cycloheximide and monitored protein levels by western blotting. Our data demonstrate that unphosphorylated Ssk1 was quickly degraded during exponential growth and after osmotic stress but remained remarkably stable during glucose limitation. We conclude that glucose starvation induces a delay in the turnover of unphosphorylated Ssk1, which is sufficient to activate the Hog1 MAPK pathway. Although unphosphorylated Ssk1 is known to be degraded by the proteasome, its stabilization is apparently not due to changes in cellular localization or decrease in ubiquitination levels during glucose limitation.

## Introduction

Yeast cells sense the levels of external nutrients through a network of signaling pathways, which control changes in metabolic rates and transcriptional profiles. Yeast prefers glucose and fructose over other carbon sources and favours fermentation over oxidative phosphorylation to fuel biosynthetic pathways with energy and precursor molecules. As a consequence, yeast cells change their transcriptional programs rapidly and globally upon changes in glucose levels.

Adaptation to environmental stress in yeast requires specific mitogen-activated protein kinase (MAPK) cascades. Five MAPK pathways have been characterized from yeast. These pathways can be activated by different stimuli and several MAPKs use overlapping upstream activation elements [1]. How a unique signaling output is triggered by a specific stimulus is not well understood and how integration of MAPK signaling with other regulatory networks is achieved remains to be resolved. The high osmolarity glycerol (HOG) MAPK cascade is a well-characterized response to osmotic stress. However, activation of the Hog1 MAPK (a homolog of mammalian p38 MAPK) is also observed in response to other physical-chemical stresses such as heat shock [2], cold stress [3], hypoxia [4]. Hog1 is therefore considered a prototype for the family of stress-activated protein kinases [5].

**Fig 1:**
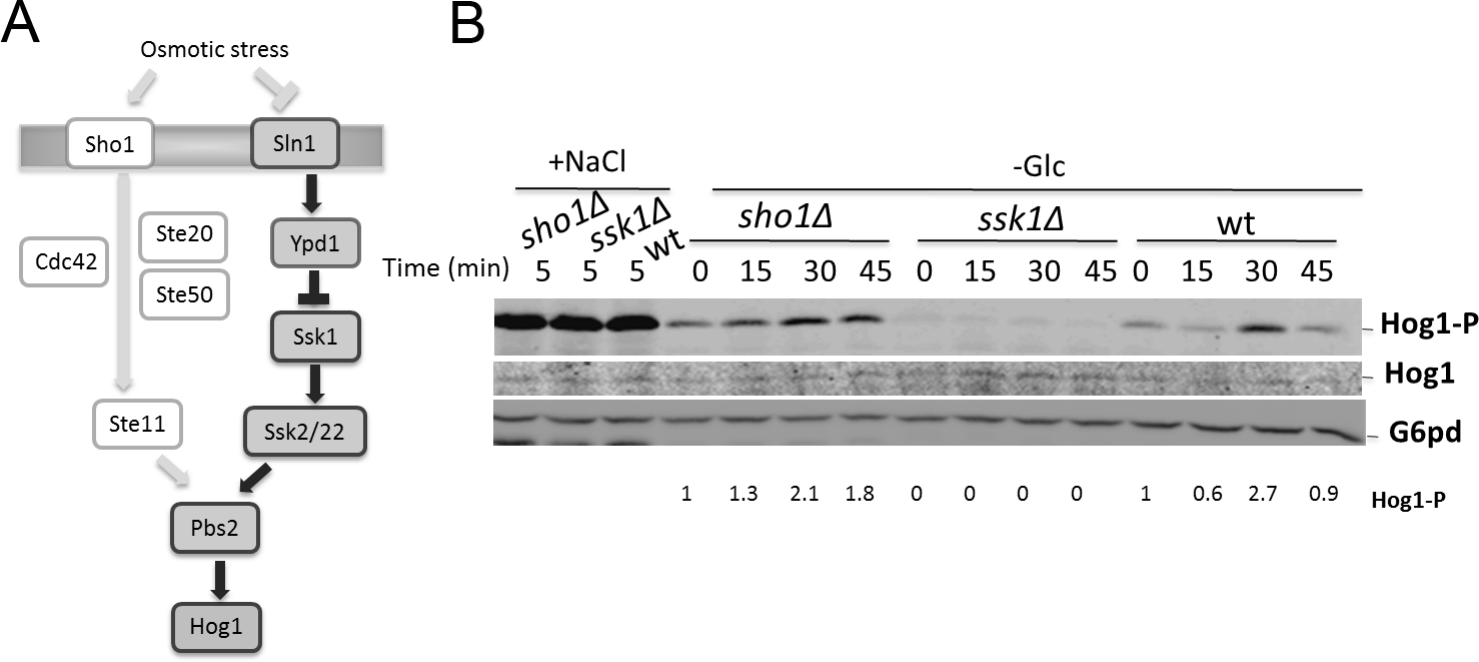
Activation of Hog1 MAPK during glucose starvation is dependent of Ssk1. (A) Schematic diagram of the yeast Hog1 pathway—Osmotic stress can be transduced by Sho1 and Sln1 activation branches. Proteins important for Hog1 phosphorylation during glucose starvation are highlighted. (B) Wild-type (wt), *ssk1*Δ and *sho1*Δ strains were exponentially grown and subjected to 0.4 M NaCl for 5 min (+NaCl) or starved for glucose for 45 min (-Glc). Cells were collected at the indicated times and equal amounts of cell extracts were analyzed by SDS-PAGE and by immunoblotting using anti-phospho-p38 antibody. Glucose-6-phosphate dehydrogenase (G6pd) was probed as the loading control. Levels of phosphorylated Hog1 were normalized using G6pd levels and then the relative quantification was normalized by time 0.

The HOG pathway is controlled by two separate upstream activation mechanisms (Fig 1A). Sln1 and Sho1 are distinct osmosensors that can independently mediate activation of Hog1 [6]. Both systems converge at the level of MAPKK Pbs2 that also functions as a scaffold protein [6]. The Sho1 branch requires the factors Ste20, Cdc42, Ste11 and Ste50 for transmitting the osmotic stress signal [6]. Ste11 and Ste50 are also part of other MAPK cascades required for filamentous growth and mating [1]. Signaling specificity is controlled by pathway-specific scaffold proteins and cross-pathway inhibition [1]. The Sln1 branch is a phosphorelay system consisting of Sln1, Ypd1 and Ssk1 [7].

We recently reported that Hog1 activation also occurs as a response to metabolic stress, specifically to acute changes in glucose levels [8]. We found that glucose deprivation triggers robust activation of Hog1, which is independent of the osmotic stress-induced activation. This novel glucose-specific control of Hog1 is required for coordinating distribution of lipid phosphatase Sac1 between ER and Golgi in response to metabolic stress, which is important for coordinating secretion with cell proliferation [8]. Importantly, this mechanism is evolutionarily conserved and the mammalian homolog p38 MAPK plays a similar role in controlling Sac1 localization and lipid signaling at the Golgi [9].

Activation of Hog1 regulates the activity of several proteins, such as transcription factors [10], cell cycle regulators [11] and metabolic enzymes [12]. However, glucose-specific Hog1 targets and how Hog1 MAP kinase is activated during carbon stress is unknown. In this work, we show that stabilization of Ssk1 is involved in the mechanism of activation of Hog1 pathway during glucose starvation in *Saccharomyces cerevisiae*.

## Materials and Methods

### Strains, plasmid and other procedures

All strains used in this study are listed in Table 1. Yeast complete media (YPD) and Hartwell’s complete media (HC) were prepared as described in [13]. For expressing Myc-Ssk1, a tag composed of three Myc epitopes were fused to the N-terminal of Ssk1. The relevant construct contained a *PstI* site upstream of the Myc epitope and a *XhoI* site downstream of the *SSK1* stop codon. The PCR-generated *PstI*/*XhoI* fragment (*myc-SSK1*) was cloned into pRS415, a shuttle vector containing *ADH* promoter and *CYC1* terminator (plasmid pMV06). To generate a *SSK1-GFP* construct, a *PstI* site was introduced upstream of the *SSK1* start codon and a *HindIII* site was introduced in the 3’ end of *SSK1* ORF. The *GFP* fragment was cloned in frame into the *HindIII* site and an *XhoI* site was introduced downstream of the *GFP* stop codon. A *PstI*/*XhoI* fragment containing *SSK1-GFP* was cloned into pRS415 containing *ADH* promoter (pMV08). Disruption of genes was performed according to standard techniques [13]. The disruption cassette was created by PCR, using primers with ends homologous to the gene to be disrupted and a vector with a selectable marker as template. The PCR product was transformed into ATY201 strain. Antibodies against Myc epitope and glucose-6-phosphate dehydrogenase (G6pd) were purchased from Sigma, anti-phospho-p38 antibody (p38-P) from Cell Signaling, and anti-Ubiquitin from Santa Cruz. Restriction enzymes were purchased from New England BioLabs. Phos-tag Acrylamide was purchased from Wako. Calf intestinal alkaline phosphatase (CIP) was obtained from New England BioLabs. Dephosphorylation reactions were conducted in standard buffer (50 mM potassium acetate, 20 mM Tris-acetate, 10 mM magnesium acetate, 100 μg/ml BSA, pH 7.9) at 37°C for 30 min.

**Table 1:** Yeast strains.

Strain	Genotype	References
ATY201	*MATalpha trp1-Δ901 leu2–3*,*112 his3-Δ200 ura3–52 lys2–801 suc2-Δ9 can1*::*hisG*	[8]
HPY138	*MATa trp1-Δ901 leu2–3*,*112 his3-Δ200 ura3–52 lys2–801 suc2-Δ9 can1*::*hisG sho1Δ*::*TRP1*	[8]
HPY127	*MATa trp1-Δ901 leu2–3*,*112 his3-Δ200 ura3–52 lys2–801 suc2-Δ9 can1*::*hisG ssk1Δ*::*TRP1*	[8]
MVY02	*MATalpha trp1-Δ901 leu2–3*,*112 his3-Δ200 ura3–52 lys2–801 suc2-Δ9 can1*::*hisG hog1Δ*::*HIS sln1Δ*::*URA*	This work

### Degradation monitoring of Myc-Ssk1

Cells expressing Myc-Ssk1 were grown in Hartwell’s complete media minus L-leucine (HC-Leu) overnight at 30°C and diluted in the morning to an OD_600_ ~ 0.1. Degradation monitoring of Myc-Ssk1 was performed in cells in early log phase (OD_600_ ~ 1) as described previously [14]. Twenty-five milliliters of culture at OD_600_ = 1.0 were concentrated in 5 ml of HC, HC without glucose (HC-Glc) or HC containing 0.4 M NaCl (HC+NaCl). Cycloheximide (0.5 mg/mL) was added to the media to stop protein synthesis. Aliquots of 1 ml (OD_600_ = 5.0) were collected at time points 0, 30, 60 and 90 minutes after addition of cycloheximide and followed by protein extraction as described in [13]. Protein levels were then analyzed by immunoblotting with anti-Myc antibody.

### Proteasome inhibition

Proteasome inhibition experiments were performed as described elsewhere [15]. Cells expressing Myc-Ssk1 were grown overnight in HC-Leu (0.17% yeast nitrogen base without ammonium sulfate) supplemented with 0.1% L-proline and 2% glucose as the carbon source. Cultures were diluted to an OD_600_ ~ 0.1 and when OD_600_ ~ 0.5, 0.003% of SDS was added and incubated for 3 hours. The proteasome inhibitor MG132 (Sigma) was solubilized in DMSO and added to final concentration of 50 μM for 30 min before addition of 0.5 mg/mL cycloheximide. Aliquots were collected at time point 0 and 30 min after addition of cycloheximide, and protein levels were analyzed by immunoblotting with specific antibodies.

### Preparation of yeast protein extracts and Immunoblotting

Yeast proteins were extracted as described previously [13] and subjected to electrophoresis in a 7% Laemmli’s SDS-polyacrylamide gel or in 7% Phos-tag gel containing 50μM Phos-tag acrylamide and 100 μM MnCl_2_. At pH 5–8, Phos-tag acrylamide forms a complex with anionic substituents in the presence of manganese or zinc ions, particularly phosphoric esters with a single ester bond. Affinity for phosphate anions is much higher than other negative ions making binding selective for phosphate groups thus decreasing the migration speed of phosphorylated proteins in SDS-PAGE. After transferring proteins to nitrocellulose membranes, nonspecific interactions were blocked with 5% non-fat milk in PBS or to monitor phosphorylation of Hog1, with 3% BSA for 2 h at room temperature. Primary antibodies anti-Myc (working dilution 1:4,000), anti-phospho-p38 antibody (working dilution 1:2,000) were incubated overnight at 4°C. After three washes with PBS-tween 0.05% was followed by 2-h incubation with secondary antibody Alexa Fluor 780 goat anti-mouse (working dilution 1:10,000) or Alexa Fluor 680 goat anti-rabbit (working dilution 1:10,000). To confirm equivalent loading, blots were reprobed with anti-glucose-6-phosphate dehydrogenase (G6pd, working dilution 1:10,000). The reactions were visualized using an Odyssey Infrared Imaging System (LIOCOR), and proteins quantification was done with ImageJ software.

### Immunoprecipitation of Myc-Ssk1

Wild-type cells transformed with vector control (pRS415) or with plasmid expressing Myc-Ssk1 (pMV06) were grown in Hartwell’s complete media minus L-leucine (HC-Leu) overnight at 30°C and diluted in the morning to an OD_600_ ~ 0.1. 50 ml of culture at OD_600_ ~ 1.0 were left in HC-Leu or washed twice in HC-Leu-Glc and incubated for 30 min in HC-Leu-Glc. Cells were collected by centrifugation, suspended in lysis buffer (100 mM Tris-HCl [pH 7.5], 1 mM EDTA, 10% glycerol, 75 mM NaCl, 0.05% SDS, 0.1% Triton X-100, protease inhibitor cocktail (ROCHE) and 10 mM N-ethylmaleimide as deubiquitinase inhibitor) and glass bead lysis. Cell debris was pelleted by centrifugation for 15 minutes 15000 g at 4°C temperature and supernatant was incubated with anti-Myc antibody at 4° C overnight and then with Protein G agarose for 2 h at 4°C. Beads were collected and washed three times with lysis buffer and then suspended in 2X Laemmli sample buffer and boiled for 5 minutes. Samples were subjected to electrophoresis in 4–15% gradient gel (BioRad) and western blotting was performed as described above with anti-Ub antibody (working dilution 1:1,000). To confirm equivalent loading, blots were reprobed with anti-Myc antibody.

### Ssk1-GFP and Hog1-GFP localization

Localization of Ssk1-GFP and Hog1-GFP were performed as described [8]. Overnight cultures of cells expressing Ssk1-GFP and Hog1-GFP were diluted in the morning to an OD_600_ ~ 0.1 in HC-Leu with 1μg/mL of DAPI and grown for three doubling times to early log phase (OD_600_ ~ 0.8–1.0). Aliquots of cell were left in HC-Leu or washed once in HC-Leu without 2% glucose (HC-Leu-Glc) and incubated for 30 min in HC-Leu-Glc or submitted to osmotic stress with 0.4 M NaCl (HC+NaCl) for 5 min. GFP and DAPI signals in living yeast cells were viewed using a Nikon fluorescence microscope (model E800) equipped with a Plan-Apo 100x/1.4 oil objective and a Photometrics Coolsnap HQ camera. Images were analyzed using Metamorph software (Universal Imaging Corporation).

## Results

We recently reported that glucose starvation induces the phosphorylation of Hog1 [8]. The starvation-induced activation of Hog1 is different from osmotic stress during which the activation of the Hog1 pathway can be transduced by both Sho1 and Sln1 branches [6] (Fig 1A). In contrast, the phosphorylation of Hog1 during glucose starvation is solely dependent on Ssk1 (Fig 1B) and independent of the Sho1 branch or the upstream osmosensing factor Sln1 [8]. During high osmolarity, the phosphorylation of Hog1 reaches a maximum within 5 minutes of treatment in either wild-type, *sho1Δ* and *ssk1Δ* cells. Hog1 activation during carbon stress is a slower process and only reached a maximum at 30 minutes after depletion of glucose (Fig 1B).

**Fig 2:**
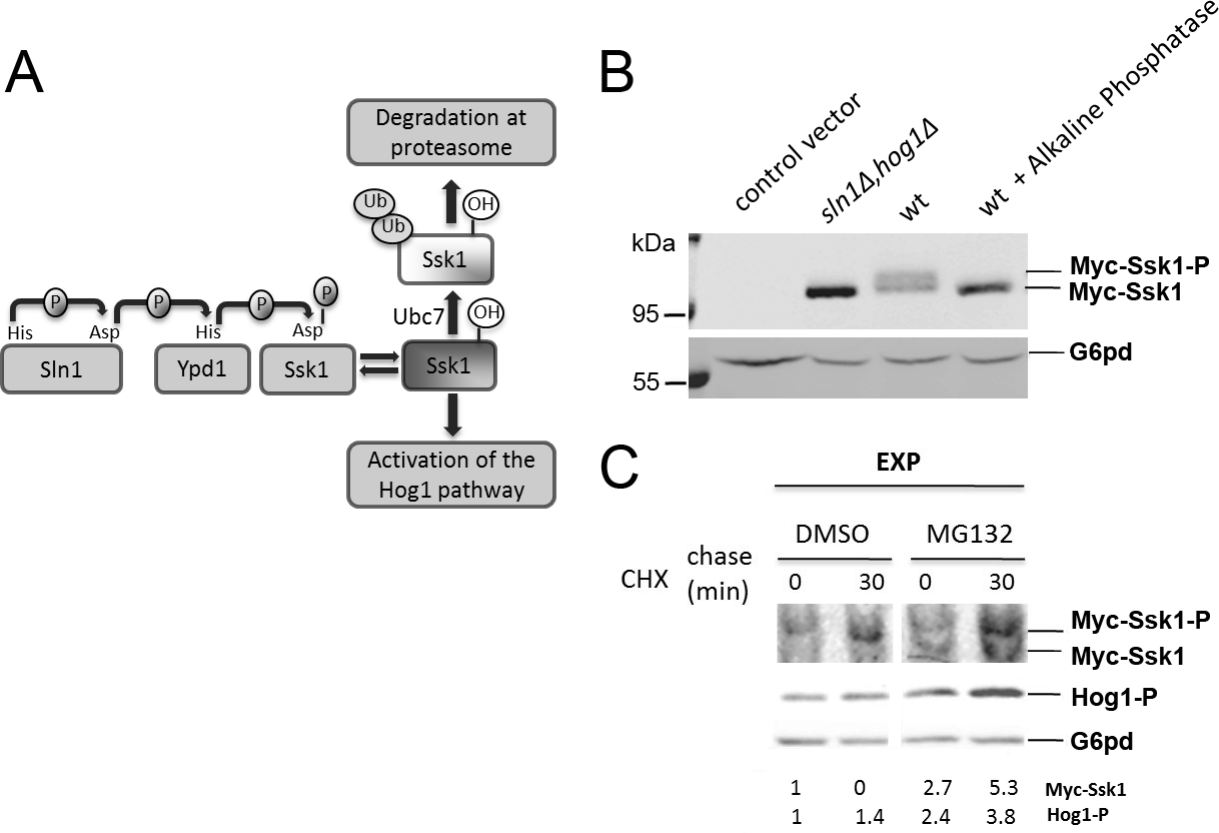
Detection of phosphorylated and unphosphorylated Ssk1. (A) Schematic diagram of Sln1 branch. Under normal osmotic conditions, Sln1 is autophosphorylated by an intrinsic His kinase activity. The phosphate group is transferred to Ssk1 via a phosphorelay system that involves the transfer protein Ypd1. Phosphorylated Ssk1 is unable to bind Ssk2/Ssk22 and keeps the pathway inactive. During osmotic stress Sln1 kinase activity is inhibited and unphosphorylated Ssk1 activates the Hog1 pathway and is ubiquitinated via Ubc7 and degraded by the proteasome. (B) Wild-type (wt) and sln1Δhog1Δ cells transformed with Myc-Ssk1 plasmid (pMV06) or wt cells containing the pRS415 vector (control) were grown as indicated. Cell extracts were analyzed by SDS-PAGE followed by immunoblotting with anti-Myc antibody. The presence of phosphorylated Ssk1 (Myc-Ssk1-P) was determined by shift of electrophoretic mobility after treatment with alkaline phosphatase CIP performed according to manufacturer’s instructions. Glucose-6-phosphate dehydrogenase (G6pd) was detected as the loading control. (C) Effects of proteasome inhibition by MG132 on degradation of Myc-Ssk1 and phosphorylation of Hog1. Wild-type yeast cells expressing Myc-Ssk1 were exponentially grown (EXP) and treated with the proteasome inhibitor MG132 or DMSO (control), containing cycloheximide to stop protein synthesis. The degradation of unphosphorylated Ssk1 was analyzed on 7% Phos-tag gel containing 50μM Phos-tag acrylamide followed by immunoblotting with anti-Myc antibody. Levels of phosphorylated Hog1 were analyzed on normal SDS-PAGE followed by immunoblotting with anti-phospho-p38 antibody. Glucose-6-phosphate dehydrogenase (G6pd) was detected as the loading control. Levels of phosphorylated Hog1 and unphosphorylated Ssk1 were normalized using G6pd levels and then the relative quantification was normalized using the DMSO sample at time 0.

Since Hog1 phosphorylation during glucose limitation is dependent on Ssk1, we expressed Ssk1 fused to a 3xMyc-tag at the N terminus under control of the ADH promoter to investigate the mechanism of activation of this pathway. Sln1 kinase transfers the phosphate group to Ssk1 at Asp554 via a phosphorelay system that involves the transfer protein Ypd1 (Fig 2A). In wild-type cells, the presence of phosphorylated Ssk1 (Ssk1-P) and unphosphorylated Ssk1 (Ssk1) was confirmed by a shift in electrophoretic mobility after treatment with alkaline phosphatase CIP at 37°C for 30 min (Fig 2B). Phosphorylated Ssk1 is in an inactive conformation, unable to bind to Ssk2/Ssk22, which keeps the pathway inactive. Elimination of Sln1 abrogates phospho-transfer to Ssk1 and causes constitutive activation of Hog1. In certain yeast strains, Sln1 deletion is lethal [16]. In our strain background a *sln1*Δ mutant is viable but grows significantly slower than the wild type [8] as a consequence of constitutive activation of Hog1. To reverse the growth phenotype of *sln1*Δ we expressed Myc-Ssk1 in a *sln1*Δ*hog1*Δ background. In *sln1*Δ*hog1*Δ mutant cells only unphosphorylated Ssk1 was present as expected (Fig 2B).

During osmotic stress Sln1 kinase activity is inhibited and unphosphorylated Ssk1 accumulates to activate the Hog1 pathway (Fig 2A). Sato et al. showed that Ssk1 is rapidly ubiquitinated via Ubc7 an E2 ubiquitin-conjugating enzyme and degraded by the proteasome [14]. In *rpn9Δ* and *rpn12–1Δ* cells (mutants defective in a 19S regulatory subunit of proteasome) Ssk1 is more stable than in wild-type cells [14]. When we expressed Myc-Ssk1 in wild-type yeast cells treated with the proteasome inhibitor MG132 the degradation of Ssk1 was inhibited resulting in increased phosphorylation of Hog1 (Fig 2C), which confirms the importance of Ssk1 turnover in the timing of Hog1 signaling.

**Fig 3:**
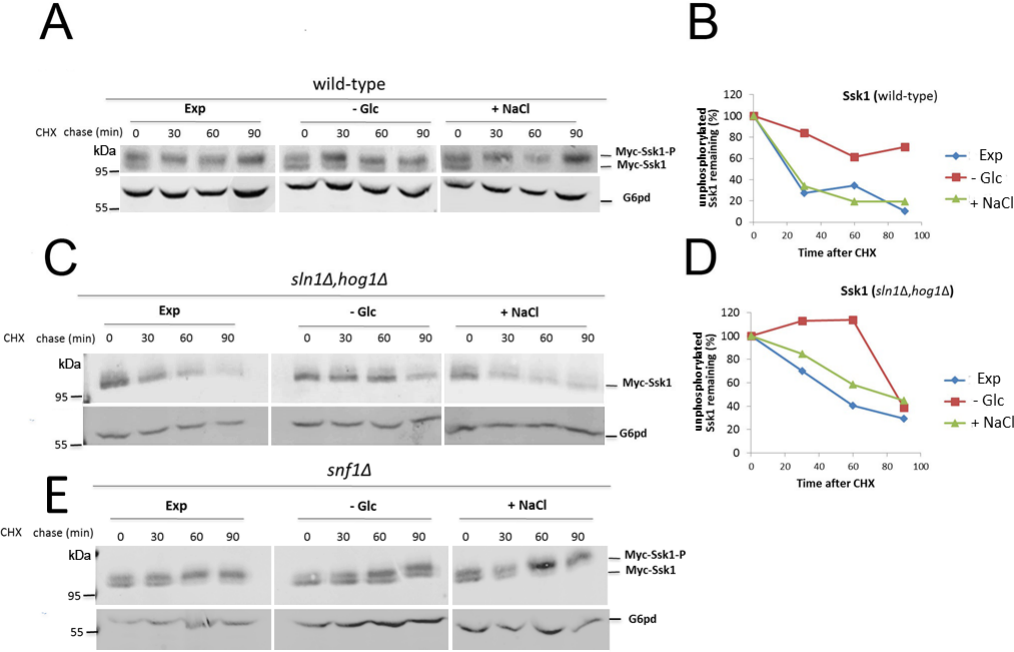
Ssk1 is stabilized during glucose starvation. Wild-type (A) and *sln1Δhog1Δ* (C) cells expressing Myc-Ssk1 were exponentially grown in HC-Leu (EXP), and then starved for glucose (-Glc) or subjected to osmotic stress (+NaCl). Cycloheximide was added to stop protein synthesis and cells were collected at the indicated times. Levels of unphosphorylated Ssk1 were monitored using SDS-PAGE followed by immunoblotting with anti-Myc antibody. Glucose-6-phosphate dehydrogenase (G6pd) was detected as the loading control. The relative quantities of unphosphorylated Ssk1 in wild-type (B) and *sln1Δhog1Δ* cells (D) were normalized using G6pd levels. E—*snf1Δ* cells expressing Myc-Ssk1 were exponentially grown in HC-Leu (EXP), and then starved for glucose (-Glc) or subjected to osmotic stress (+NaCl). Cycloheximide was added to stop protein synthesis and cells were collected at the indicated times. Levels of unphosphorylated Ssk1 were monitored using SDS-PAGE followed by immunoblotting with anti-Myc antibody. Glucose-6-phosphate dehydrogenase (G6pd) was detected as the loading control.

Since the activation of Hog1 during glucose starvation is dependent on Ssk1 and stabilization of Ssk1 induces the phosphorylation of Hog1, we examined the turnover of Ssk1 upon glucose starvation in the presence of cycloheximide and monitored protein levels by western blotting. In wild-type cells, the amount of Ssk1-P and Ssk1 was similar at time 0 after cycloheximide addition in exponentially growing cells and in cells subjected to osmotic stress or glucose limitation (Fig 3A). The presence of some unphosphorylated Ssk1 in the absence of specific stress conditions is likely related to base level activity of the pathway and there are several mechanisms that prevent incorrect activation of Hog1 [17]. In wild-type yeast cells the Ssk1-P form was more stable than Ssk1 during exponential growth and osmotic stress. Ssk1 was quickly degraded during exponential growth and osmotic stress but remained remarkably stable during glucose limitation (Fig 3A and 3B). These results indicate that turnover of unphosphorylated Ssk1 is delayed in glucose starved cells, which in turn promotes Hog1 phosphorylation.

To investigate the possible involvement of Ssk1-P in the stabilization of Ssk1 during glucose starvation we monitored levels of Ssk1 in *sln1Δhog1Δ* cells. In this mutant we observed similar results as in wild-type cells (Fig 3C and 3D). Ssk1 is quickly degraded during exponential growth and osmotic stress and stabilized during glucose limitation. This result indicates that the stabilization of Ssk1 during glucose starvation was independent of the phosphorylated Ssk1 isoform.

It is known that activation of Snf1, the yeast homolog of mammalian AMP-dependent kinase (AMPK), is an important response to glucose starvation in yeast via regulating the expression of genes necessary to utilize others carbon sources [18]. Our previous results indicated that Snf1 may participate in Hog1 activation during starvation [8]. To investigate the possible involvement of Snf1 in delaying the turnover of Ssk1, we monitored the levels of Ssk1 in *snf1Δ* mutants. However, our results show that Ssk1 is stable in *snf1Δ* cells during carbon stress, indicating that AMPK is not critical for the metabolic regulation of Hog1 (Fig 3E)

**Fig 4:**
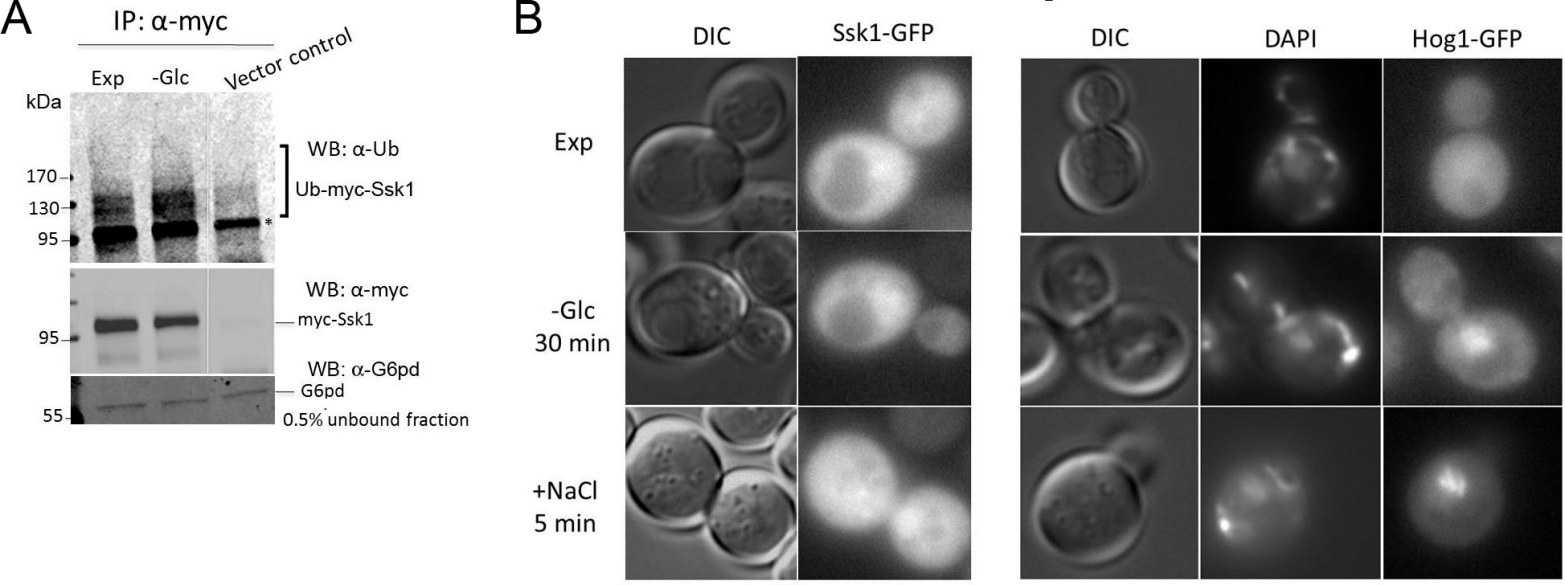
Ubiquitination and localization of Ssk1. (A) Wild-type cells transformed with vector control (pRS415) or with a plasmid expressing Myc-Ssk1 were exponentially grown in HC-Leu (Exp), and subjected to glucose starvation for 30 min (-Glc). Myc-Ssk1 was immunoprecipitated with anti-Myc antibody and ubiquitinated Ssk1 was detected by Western blotting using anti-Ub antibodies. Equal loading of each lane was confirmed by reprobing with anti-Myc antibodies and by immunoblotting with anti-G6pd antibodies. * nonspecific band. (B) Localization of Ssk1-GFP. Wild-type cells expressing Ssk1-GFP were exponentially grown in HC-Leu (EXP), and then starved for glucose for 30 min (-Glc) or subjected to osmotic stress (+NaCl) for 5 min. A strain expressing Hog1-GFP was used as positive control. Intracellular localization of Ssk1-GFP and Hog1-GFP was determined by fluorescence microscopy.

Post-translational modification by ubiquitination regulates the quantity of Ssk1 present in the cytoplasm and consequently the extent of Hog1 MAPK pathway activity. Ubiquitination requires the coordinated action of three enzymes: ubiquitin-activating enzyme (E1), ubiquitin-conjugating enzyme E2 (Ubc7) and E3 ubiquitin ligase, which provides substrate specificity. The ubiquitin ligase E3 responsible for Ssk1 ubiquitination has yet to be identified [14]. To investigate the possibility that stabilization of Ssk1 and activation of Hog1 during glucose starvation is due to decreased activity of proteins responsible for Ssk1 polyubiquitination (E1-E3), we examined levels of ubiquitinated Ssk1 by western blotting using an anti-Ub antibody. As shown in Fig 4A, ubiquitinated Ssk1 was detected in both conditions, during exponentially growth and glucose starvation. This result indicates that the process of ubiquitination is not diminished during glucose stress. Additionally, during glucose stress the amounts of ubiquitinated Ssk1 seemed to be higher. This increase was expected because Ssk1 is more stable during starvation and ubiquitinated Ssk1 accumulating in the cytoplasm appears to be functional for activating the Hog1 pathway.

It is known that the degradation of Ssk1 is dependent on the E2 ubiquitin-conjugating enzyme Ubc7, which is located at the endoplasmic reticulum [14, 19]. Furthermore, signaling pathways are often regulated by subcellular localization and compartmentalization of signaling molecules [20]. We therefore asked whether the stabilization of Ssk1 during glucose stress is related to Ssk1 sequestration at specific intracellular sites, thus preventing contact with Ubc7. To examine this possibility, the localization of Ssk1 was monitored by using GFP fused in frame at the C-terminus of the Ssk1 protein under control of the ADH promoter. Wild-type cells expressing Ssk1-GFP were exponentially grown, and then starved for glucose during 30 min or subjected to osmotic stress for 5 min. As shown in Fig 4B, Ssk1 is located at the cytoplasm and nucleus during normal growth conditions and there is no apparent change in the subcellular localization of Ssk1 upon osmotic stress or during glucose limitation. A strain expressing Hog1-GFP was used as a control, showing that Hog1 translocates to the nucleus during osmotic and carbon stress as expected [8, 21].

## Discussion

We have recently demonstrated that the Hog1 MAPK pathway in yeast is regulated by glucose availability [8]. Similarly as in osmotic stress, glucose starvation leads to a transient accumulation of Hog1 in the nucleus [8]. However, the kinetics and the mechanism of Hog1 activation are substantially different. During osmotic stress the activation of Hog1 can be transduced by both Sho1 and Sln1 branches and during glucose starvation the phosphorylation of Hog1 is slower and is completely dependent on Ssk1 [8]. In this work, we elucidated parts of the mechanism of activation of the Hog1 pathway during carbon stress. Our data demonstrate that glucose deprivation induces a delay in the turnover of unphosphorylated Ssk1 which is sufficient to activate the Hog1 MAPK pathway. Although Ssk1 is known to be degraded by the proteasome [14], its stabilization is apparently not due to changes in cellular localization or even decrease in ubiquitination levels during glucose limitation.

Although the precise mechanism of a delay in Ssk1 turnover during carbon stress remains unknown, several possibilities can be considered. For example proteasome activity may be downregulated during acute glucose depletion resulting in Ssk1 stabilization and thus result in activation of Hog1 MAPK. In fact, recent evidence shows that in starved quiescent yeast cells, proteasome subunits relocate from the nucleus to cytoplasm and assemble in a structure called proteasome storage granules (PSGs) that are thought to protect the proteasome from autophagic degradation [22, 23]. However, proteasome relocation was observed after 24 hours in glucose-depleted media [22, 23] and it is unclear whether this effect is an early response to starvation and accountable for the relatively fast stabilization of Ssk1. Another possibility is the regulation of Ssk1 turnover via an unknown factor during glucose depletion, which in turn promotes Hog1 activation. Snf1 AMPK, a central regulator of the cellular response to glucose starvation could play such a role [18]. However, our results show that Ssk1 stabilization during carbon stress is independent of Snf1, ruling out a major role of this pathway in controlling Hog1 at the level of Ssk1.

During the osmotic stress response, Hog1 has both transcriptional and cytoplasmic roles that help yeast cells to adapt to changes in environmental conditions [24, 25]. Although glucose-specific Hog1 targets are unknown, our previous work established that Hog1 is required for coordinating the retrograde traffic of Sac1 lipid phosphatase to the ER in response to acute changes in glucose levels, which is important for regulating the levels of phosphatidylinositol-4-phosphate (PI4P). Thus, Hog1 is involved in the control of membrane trafficking during cell proliferation in yeast [8].

More work will be necessary to define the precise mechanism of Ssk1 stabilization and consequently Hog1 activation during glucose limitation. Understanding the details of the metabolic regulation of Hog1 may help to discover novel drug targets since Hog1 is a homolog of mammalian p38 MAPK, which plays important roles in the inflammation process [26] and cancer [27].
